# Electro-Magnetism in Acute Diseases

**Published:** 1873-02

**Authors:** A. Donald

**Affiliations:** Louisiana


### ELECTRO-MAGNETISM IN ACUTE DISEASES.

BY A. DONALD, M.D., OF LOUISIANA.

We are aware that our theory of what is called *vital force* has many objectors, even among very high authorities. We are not responsible for any man's objections, nor shall we stop to answer many of them. It is objected by some that electro-magnetism is not the vital force in the animal economy, because it has not the attribute of sensation. We do not understand that vital force is sensation; but we can understand how it should be the medium, and *a part of the cause*, of sensation. The objector says that electro-magnetism only serves to arouse vital force. Let us see if this objection will stand the test of analysis. *Vital force is not inactive; it is a force already in action;* otherwise, it is no force. If a force, then *it needs no help.* Thus, the objection fails. Hereafter, we may have something to say about the laws and cause of sensation.

But vital force, like all other forces, must be governed by some law. It is not a *lawless force, running about at random.* The Creator of the Universe never left any of His works in this reckless condition. The human pulse is the representative idea of the vital force, which accords in its action to the laws of electro-magnetism, or positive and negative. Thus, we reach the cause of the vital force, which has its function. It is not sensation or intelligence, but contributes to sensation and intelligence. The vital force, then, belongs to certain *structural* arrangements, whose machinery, in all its perfection, we refer to the Infinite Mind; but, to our mind, no mystery attaches to the phenomena of the vital force. It is an electro-magnetic action. Mental force, or the force belonging to the element mind or soul, is a different force from that which we denominate vital force.

Having said "*thus much*" about vital force, we now propose to show how our philosophy works in establishing the functional harmony of certain pathological conditions, known as congestions, irritations and inflammations. If, for example, we take a metallic *capillary tube*, and force it full of water, and then suspend it in a vertical position, the water, if it passes, will only do so *very slowly*, in drops; but now, if we pass a current of electricity through this tube, the water will pass out in a stream. In local congestions of the capillary blood vessels, the same result will follow, as we have often demonstrated at the bedside. The following case will serve to illustrate our position:

In the fall of 1860, we were called to see Mr. M., a gentleman about fifty-five years old, who, we learned, had been on what is sometimes called "a big drunk" for about two weeks. We found him much prostrated from excessive vomiting. He could not retain a teaspoonful of water one moment, in consequence of the congestion and irritation of his stomach. There was also much pain in the head, and the pulse was unsteady, with an incessant demand for water. I sent to my office for my battery. As soon as I put it in operation, and he heard the noise, he ordered that "that damned rattle-snake be taken out of the room." I assured him that it had "no fangs," and would not hurt him; and I then told him to be quiet, and I would soon allow him as much water as he wanted, and that he could not vomit it up. Being thus assured, he permitted me to apply it. I placed one pole on the back of his neck, and took the other pole in my left hand and made the connection by carrying my right hand over his stomach, and in about twenty minutes I told him he might have half a tumbler of water; he drank it. *I did not remove* my hand, but continued the current. He did not vomit, and he continued to take water about every twenty minutes until he was satisfied. I continued the electricity for about three hours without intermission, and when I stopped, the *congestion* and *irritation* were relieved; he passed into a quiet sleep, and from that time (about twelve o'clock at night) he rested well until morning, when he arose perfectly sober, having no disposition to renew his spree, but ordered his carriage and horses, and, with his wife, went on his way home rejoicing.

Thus, it will be seen that, in this *extreme case*, the application of a remedy upon the principle of "electrical vital force" cured my patient. I have repeated the same thing in many other similar cases, with the same result, and in such cases I regard it as the safest, and by far the best, of any method of which I have any knowledge.

If you should think the principle or the facts worth anything, use them; otherwise, throw them in the rubbish corner. "Go slow."

